# Rectus Femoris Mimicking Ultrasound Phantom for Muscle Mass Assessment: Design, Research, and Training Application

**DOI:** 10.3390/jcm10122721

**Published:** 2021-06-20

**Authors:** Nobuto Nakanishi, Shigeaki Inoue, Rie Tsutsumi, Yusuke Akimoto, Yuko Ono, Joji Kotani, Hiroshi Sakaue, Jun Oto

**Affiliations:** 1 Department of Disaster and Emergency Medicine, Graduate School of Medicine, Kobe University, 7-5-2 Kusunoki, Chuo-ward, Kobe 650-0017, Japan; inoues@med.kobe-u.ac.jp (S.I.); windmill@people.kobe-u.ac.jp (Y.O.); kotanijo@med.kobe-u.ac.jp (J.K.); 2 Emergency and Critical Care Medicine, Tokushima University Hospital, 2-50-1 Kuramoto, Tokushima 770-8503, Japan; ysk807@tokushima-u.ac.jp (Y.A.); joto@tokushima-u.ac.jp (J.O.); 3 Department of Nutrition and Metabolism, Tokushima University Graduate School of Biomedical Sciences, 3-18-15 Kuramoto, Tokushima 770-8503, Japan; rtsutsumi@tokushima-u.ac.jp (R.T.); hsakaue@tokushima-u.ac.jp (H.S.)

**Keywords:** ultrasonography, muscles, quadriceps muscle, sarcopenia, education

## Abstract

Ultrasound has become widely used as a means to measure the rectus femoris muscle in the acute and chronic phases of critical illness. Despite its noninvasiveness and accessibility, its accuracy highly depends on the skills of the technician. However, few ultrasound phantoms for the confirmation of its accuracy or to improve technical skills exist. In this study, the authors created a novel phantom model and used it for investigating the accuracy of measurements and for training. Study 1 investigated how various conditions affect ultrasound measurements such as thickness, cross-sectional area, and echogenicity. Study 2 investigated if the phantom can be used for the training of various health care providers in vitro and in vivo. Study 1 showed that thickness, cross-sectional area, and echogenicity were affected by probe compression strength, probe angle, phantom compression, and varying equipment. Study 2 in vitro showed that using the phantom for training improved the accuracy of the measurements taken within the phantom, and Study 2 in vivo showed the phantom training had a short-term effect on improving the measurement accuracy in a human volunteer. The new ultrasound phantom model revealed that various conditions affected ultrasound measurements, and phantom training improved the measurement accuracy.

## 1. Introduction

Skeletal muscle is important to surviving critical illnesses [1]. At the intensive care unit (ICU) admission, higher muscle mass is associated with decreased mortality [2]. After the ICU admission, progressive muscle atrophy occurs, and the atrophy is associated with ICU acquired weakness and mortality [3]. Every 1% loss of muscle mass is associated with 5% higher odds ratio of 60-day mortality in critical illness [4]. In the chronic phase, 73% of patients demonstrated persistent muscle atrophy at 6 months after ICU discharge [5]. Furthermore, muscle atrophy progresses 6 to 12 months after ICU discharge [6].

Muscle mass assessment is important to manage nutrition and rehabilitation [7]. Although numerous ways to measure muscle mass currently exist [8], ultrasound has been gaining popularity for muscle mass assessment in critically ill patients [9]. The rectus femoris muscle correlates with whole-body muscle mass [10] and physical functions [11]; thus, ultrasound assessment is often conducted at this point. Although ultrasound is known to be noninvasive and accessible, even at the bedside, accurate measurement requires a high degree of skill and experience [12]. Therefore, an urgent need for a well-established method of measurement and training is present to promote accurate measurements.

However, there are insufficient training opportunities in musculoskeletal ultrasound [13]. Ultrasound training often uses phantom models, which artificially mimic organ structures with adjusted acoustic properties. Despite the existence of cardiac or abdominal ultrasound phantom models [14,15], few ultrasound phantom models exist for the measurement of the rectus femoris muscle. Several ultrasound phantoms were created for muscle quality assessments [16,17], but not for muscle quantity assessments. As a phantom model has a predetermined muscle size, it can be used for the assessment of accurate measurements. It is important to assess how probe angle, compression strength, and a different device can affect muscle mass measurements. Moreover, predetermined echogenicity can be used to assess how various conditions change echogenicity. Furthermore, authors need to know whether measurement accuracy is improved with the use of the phantom as a training device to teach established methods. Phantom training can improve ultrasound skills and reduce the scanning time to obtain a clear ultrasound image [18]. Due to the noninvasiveness, ultrasound can be used by various levels of health care providers [19]. Therefore, ultrasound techniques are important for most health care providers, including physical therapists, nurses, and nutritionists. Thus, the creation of a training phantom is important.

In this study, the authors devised a training phantom model for measuring rectus femoris muscle mass. Using this phantom, the authors evaluated whether ultrasound measurements were affected by various measurement conditions and if this model could be used for the training of the measurement of rectus femoris muscle mass. In Study 1, the authors used the phantom model for evaluating the accuracy of thickness, cross-sectional area, and echogenicity measurements in different settings including different compression, angle, and equipment. Then, in Study 2, the authors used the phantom to train various health care providers and tested whether it would improve the accuracy of the measurements in vitro and in vivo.

## 2. Materials and Methods

### 2.1. Study Design and Settings

The authors collaborated with Kyoto Kagaku (Kyoto, Japan) to create a phantom model from February to August 2020. Then, the authors applied the phantom model to research and training purposes in Tokushima University Hospital from September 2020 to January 2021. This study was approved by a clinical research ethics committee of Tokushima University Hospital (approval number 2593). At the time of enrollment, written informed consent was obtained from the volunteers.

### 2.2. Phantom Model

**Figure 1:**
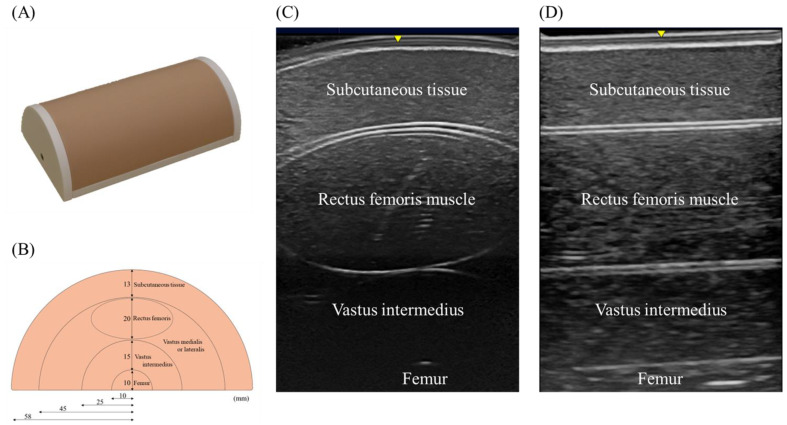
Ultrasound phantom of the rectus femoris muscle. (**A**) Phantom model image. (**B**) Cross-sectional structure of the phantom model; this phantom has a semicircle structure. From the surface of the vertical line surface, internal layers include subcutaneous tissue (13 cm), rectus femoris muscle (20 cm × 40 cm), vastus intermedius (15 cm), femur (10 cm), and at the same layer with rectus femoris muscle, vastus medialis or lateralis. (**C**) Cross-sectional ultrasound scanning image. (**D**) Longitudinal ultrasound scanning image.

The authors have created a novel phantom model of the lower limb primarily for measuring the rectus femoris muscle. The phantom is designed to be a cylindrical shape for training purposes. The size is 22.0 cm in length, 11.6 cm in width, and 6.8 cm in height (Figure 1A). The two-dimensional structure is a concentric semicircular structure (Figure 1B). The size written in the figure is that of the design, which is not exactly equivalent to the actual size. The structure contains the rectus femoris, vastus medialis, vastus lateralis, and vastus intermedius muscles, as well as the femur and subcutaneous tissue. The authors demonstrate the scanning image with a linear probe at the cross-sectional and longitudinal direction (Figure 1C,D).

This phantom is composed of urethane modified to be suitable for ultrasound scanning. The modification was conducted by adjusting acoustic sound impedance. Then, these parts were constructed in the design mentioned above. The constructed phantom was stabilized by 10 mm and 12 mm of rigid urethane foam at the lateral side and bottom, respectively. The surface of the phantom was coated with silicon. The cross-sectional image of the phantom is close to the scanning at one-third to one-half from the proximal end of the patella to the anterior superior iliac spine. Acoustic characteristics of phantom material were measured by Sing-around unit and solid measuring cell (UVM-2 and XM-0031, Cho-Onpa Kogyo, Tokyo, Japan) in cross-sectional and longitudinal section. As a result, sound speed was 1432 m/s, density 0.954 g/cm^3^, attenuation coefficient 0.59 dB/cmMHz, acoustic impedance 1.37 rayl at 25 °C. After the measurement, the material was adjusted to mimic muscle tissue about the ultrasound image and subjective compression feeling.

### 2.3. Study 1: Research Application

**Figure 2:**
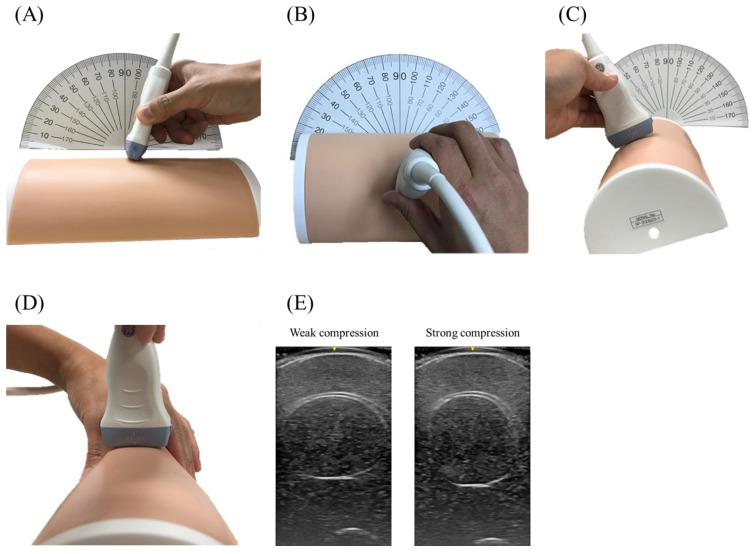
Measurements in various conditions (Study 1). The measurements were conducted in various conditions. (**A**) pennation angle change. (**B**) Horizontal angle change. (**C**) Rotation of phantom. (**D**) Compression from the side. (**E**) Ultrasound scanning image at weak or strong compression.

**Figure 3:**
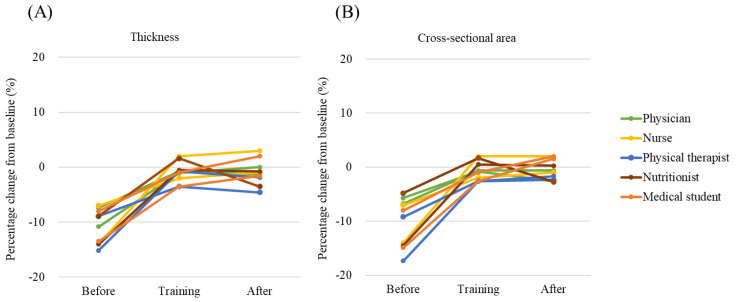
Measurement accuracy before and after the phantom training (Study 2-1). (**A**) Thickness. (**B**) Cross-sectional area. Statistical significance was tested using 95% confidence intervals (CIs), with intervals not including 0% considered as significantly inaccurate using JMP Statistical Software version 13.1.0 (SAS Institute Inc., Cary, NC, USA).

**Figure 4:**
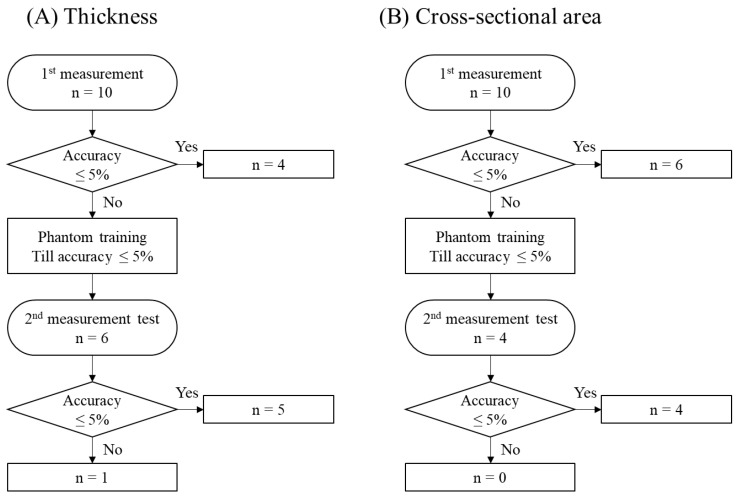
Measurement accuracy in a human volunteer (Study 2-2). (**A**) Thickness. (**B**) Cross-sectional area. Three months after the phantom training, around half of the examiners obtained the measurement accuracy within 5%. After the 2nd phantom training to obtain the measurement accuracy within 5%, almost all examiners obtained the measurement accuracy within 5%.

This study was conducted to evaluate the change in thickness, cross-sectional area, and echogenicity of ultrasound measurements in various conditions. Authors used an ultrasound with a linear probe (LOGIQ e V2 and L6-12-RS liner probe (4–13 MHz, 38.4 mm field of view, 8.0 cm depth of field), GE Healthcare Japan, Tokyo, Japan). A single examiner (N.N.) objectively measured these variables with a scale or protractor. First, the authors evaluated the baseline size and echogenicity of the rectus femoris muscle by ultrasound because the actual size did not completely reflect the original design. Thickness was measured from the upper surface of the rectus femoris muscle to the bone, and the cross-sectional area was measured by tracking the area in the transverse plane. Echogenicity was measured using Image J software version 1.51 (National Institutes of Health, Bethesda, MD, USA) after the data was transferred from the ultrasound device to a computer. In echogenicity measurements, authors determined the region of interest in the all-visible muscle area in the rectus femoris cross-sectional area with the surrounding epimysium and artifacts excluded. The mean echogenicity was calculated from the histogram of grayscale images as a value between 0 (black) and 255 (white). Then, measurements were conducted in various conditions: (1) probe compression strength (0.5 cm, 1 cm): the compression depth was checked with the ultrasound image, (2) pennation angle at 10° or 20° (Figure 2A), (3) horizontal angle at 10° or 20° (Figure 2B), (4) rotation at 20° or 40° simulating internal or external rotation of the leg (Figure 2C), (5) compression from both sides simulating the extension or flection of the knee (Figure 2D,E), (6) different probe: linear or convex (4C-RS (2–5 MHz, 66.2 mm field of view, 33.0 cm depth of field), GE Healthcare Japan, Tokyo, Japan), and (7) different ultrasound device (HI VISION Preirus and EUP-L73S liner probe (4–9 MHz, 47.0 mm field of view, 8.0 cm depth of field), Hitachi Medical Corporation, Tokyo, Japan).

In these measurements, the baseline linear probe was set to a frequency of 10 MHz, a gain of 50 dB, a dynamic range of 78, a focal depth of 3 cm, and a frame rate of 65 fps. The convex probe was set to a frequency of 4 MHz, a gain of 50 dB, a dynamic range of 72, a focal depth of 3 cm, and a frame rate of 37 fps. On the other hand, the different ultrasound device was set to a frequency of 9 MHz, a gain of 10 dB, a dynamic range of 75, a focal depth of 3 cm, and a frame rate of 31 fps. All measurements were conducted five times, and the change from the baseline values was compared in each condition. The difference between thickness and cross-sectional area was also evaluated.

### 2.4. Study 2-1: Training Purpose In Vitro

This study was conducted to evaluate whether the use of the phantom would improve ultrasound techniques. The authors included two volunteers from five different occupations including physicians, nurses, physical therapists, nutritionists, and medical students. A total of 10 volunteers, who did not have experience in measuring muscle mass, conducted the measurements of the thickness and cross-sectional area of the rectus femoris. The measurements were conducted in three stages. First, the examiners conducted the measurement by themselves without assistance. Second, the examiners conducted the measurements with the assistance of N.N. Six measurement points, which were considered to influence ultrasound measurements from Study 1 (Table 1) were taught. At these points, the compression of muscle was intentionally avoided by using generous amounts of gel and no compression. The probe position was set at a perpendicular angle, and the stabilized position was held by the contact of the examining hand on the phantom.

At this stage, inadequate measurement techniques were fixed by N.N. Third, the volunteers scanned the phantom by themselves without assistance. Each measurement consisted of 5-min intervals. The authors evaluated the percentage change from the base measurements in each stage.

### 2.5. Study 2-2: Training Purpose In Vivo

The 10 volunteers conducted the thickness and cross-sectional area measurements in a healthy subject 3 months after the in vitro training. First, a well-experienced examiner (N.N.) conducted measurements at the thickness from the upper surface of the rectus femoris muscle to the bone and the rectus femoris cross-sectional area at the midway between the anterior superior iliac spine and the proximal end of the patella in the healthy volunteer. The measurement was conducted 5 times, and the average value was used as a baseline value for comparison. Then, the 10 volunteers conducted the same measurements. If the measurements exceeded 5% difference from baseline value, examiners conducted the training with the phantom model until they can obtain measurement accuracy within 5% from baseline values in the phantom. After the in vitro training, examiners repeated the measurements in the healthy volunteer. Authors set a strict measurement accuracy of 5% for better accuracy, although within 10% measurement accuracy was used in another study [20].

### 2.6. Statistics

Continuous data were presented as mean ± standard deviation or median (interquartile range (IQR)), as appropriate. Normally distributed data were compared using the *t*-test, and non-normally distributed data were compared using the Mann–Whitney test. Statistical significance of the inaccuracy of measurements by various health care providers was tested using 95% confidence intervals (CIs), with intervals excluding 100% considered as statistically significant. Sample size and effect size were not calculated due to the exploratory nature of this study. Data analyses were conducted using JMP Statistical Software version 13.1.0 (SAS Institute Inc., Cary, NC, USA). All statistical tests were two-tailed, and a *p* value less than 0.05 was considered statistically significant.

## 3. Results

Study 1 showed that the measured thickness and cross-sectional area had differed from the baseline values in various conditions (Table 2). Deviation from the baseline was not observed in the rotation, a different ultrasound device, and a horizontal angle of 10° and 20° in the measurements of thickness. In the comparison of thickness and cross-sectional area, there was a difference at 1.0 cm in compression (*p* < 0.01), a pennation angle of 10° (*p* = 0.02) and 20° (*p* < 0.01), a horizontal angle of 10° (*p* = 0.01) and 20° (*p* < 0.01), a weak (*p* < 0.01) and strong (*p* < 0.01) compression, and a convex probe (*p* < 0.01). Echogenicity was mostly affected by various conditions except for a 10° horizontal angle (*p* = 0.60, Table 3).

Study 2-1 in vitro showed the accuracy in measurements before and after the point training (Figure 3). In the measurement of thickness, the percentage change from the baseline was −10.8% (95% CI, −13.0% to −8.5%) before training, −1.0% (95% CI, −2.3% to 0.3%) during training, and −1.0% (95% CI, −2.6% to 0.7%) after training. In the measurement of cross-sectional area, the percentage change from the baseline was −10.2% (95% CI, −13.5% to −7.0%) before training, −0.8% (95% CI, −2.0% to 0.4%) during training, and −0.5% (95% CI, −1.8% to 0.8%) after training.

Study 2-2 in vivo showed the effect of phantom training in a volunteer (Figure 4). Three months after the training, the accuracy of thickness measurement was not within 5% in 6 out of 10 participants, but 5 participants obtained the accuracy within 5% after the second phantom training. On the other hand, the accuracy of cross-sectional area was not within 5% in 4 out of 10 participants, but all 4 participants obtained the accuracy after the second training.

## 4. Discussion

In this study, the authors created a rectus femoris muscle phantom model. To the best of our knowledge, this is the first phantom model for ultrasound measurements of the rectus femoris muscle mass. The authors used this phantom model for two purposes, namely measurement accuracy and training application. Authors found that thickness, cross-sectional area, and echogenicity were affected by the measurement techniques, including compression strength, angle, and devices used. Moreover, point training using rectus femoris phantom improved the accuracy of measurements for various levels of health care providers.

In Study 1, the authors found that measurements were influenced by various conditions. Although the cross-sectional area is reportedly more reliable than thickness measurements [21,22], both were affected by measurement techniques. Both thickness and cross-sectional area were affected by only a 0.5 or 1.0 cm probe compression. Specifically, thickness measurements were more influenced by the probe compression. This result was consistent with a previous study, which reported that examiner force affected thickness measurements [23,24]. The pennation angle ambiguated the lower border of the muscle, affecting the measurement of the cross-sectional area. However, a previous study reported no significant difference was observed in <5° pennation angle at transversus abdominis thickness [25]. As this study was conducted only at 10° and 20°, less than 5° pennation angle may be permitted. Horizontal angle did not affect the thickness, but affected cross-sectional areas due to the transverse two-dimensional dissection. Essentially, ultrasound measurements need to be conducted in a supine position with limbs extended because limb flexion changes the figure of muscles. The authors simulated the flexion or extension model by using compression from both sides and confirmed that the condition greatly affected the measurements. A previous study also reported hip flexion increased the rectus femoris cross-sectional area by 11.1% from 0° to 60° bed elevation [26]. In ultrasound measurement, it is important to measure in a flat position with extended limbs. Although the rotation of the phantom simulating internal or external leg rotation did not change the measurements in this model, muscle location may change in a human subject. Moreover, it requires adequate skills to measure perpendicularly in the rotated position. As for the probe, the convex probe had different results from the linear probe. The convex probe has the advantage to scan the image in a single frame, but the large image and ambiguous border may cause differing results from the linear probe. The linear probe is the standard for rectus femoris muscle measurements [12], and it is important to know that the measurement by the convex probe may have a 1–2% discrepancy from the linear probe. Moreover, different ultrasound devices did not have different results, making it possible for a multicenter study to be conducted. Since muscle atrophy occurs 1.2–3.0% per day in clinically ill patients [27], a high discrepancy will cause substantial influence on the results. Our result indicates that accurate measurement requires an understanding of these results and sufficient training.

This phantom does not reflect the actual echogenicity of a human subject. However, the same object revealed how measurement technique affects echogenicity. The authors found that most conditions affected the echogenicity. Different pennation angles and compression from the side severely affected the echogenicity. Echogenicity progressively decreased with increased pennation angle (−23.1% to −27.7% in 10° to 20°). This result was almost consistent with a previous study (−15.1% to −34.9% in 10° to 20°) [23]. It is important to note pennation angle decreases echogenicity. Theoretically, a different horizontal angle and the measurement at rotations do not seem to influence the echogenicity if the probe is perpendicularly placed; however, the perpendicular position of the probe may be difficult at 20° of the horizontal angle and the rotated phantom. Moreover, the contact with gel or the shadow may affect the echogenicity. The probe compression or compression from the side simulating flexion or extension will aggregate the muscle component, resulting in lower echogenicity. The authors used the convex probe or a different ultrasound device. The convex probe or the different ultrasound setting was adjusted to be the same as much as possible, but the echogenicity was different due to the different equipment or settings. It is important to know that the echogenicity is easily affected by various conditions. According to previous studies, increased echogenicity may indicate atrophied muscle with increased fibrous tissue, necrotized muscle, and muscle weakness [28,29,30]; therefore, echogenicity in a unified setting may be clinically useful if the examiners are well trained.

In Study 2-1 in vitro, authors found that this phantom is useful for muscle mass measurement training. As Harris et al. reported that a simple training with feedback improved the accuracy of ultrasound measurements [31], point training with this phantom improved the accuracy of measurements. Before the training, the confidence interval of the measurements excluded 100%, suggesting measurements without training are inaccurate. In all examiners, the measurements were lower than the baseline size due to the insufficient gel and strong compression. However, the phantom training improved the measurement accuracy, which included 100% in the confidence interval. As ultrasound phantom has a predetermined muscle size, the phantom training will allow immediate real-time feedback rather than conducting the measurement on the volunteers. Importantly, this phantom improved the measurement accuracy in various health care providers. Thus, muscle mass measurement by ultrasound will be possible for various health care providers. This phantom can be used to acquire basic ultrasound skills as shown in Table 1. The hands-on practice using this phantom will ensure the accuracy of measurements.

In Study 2-2 in vivo, around half of the examiners did not have the accuracy within 5% at 3 months after the phantom training. This result indicates the phantom training may not have a long-term effect on ultrasound skills. However, after the second phantom training, almost all patients accomplished the accuracy within 5%. This result indicates phantom training has some short-term effect on ultrasound skills. This phantom can be used repeatedly until they can obtain an accurate measurement within 5%. After the training, the measurements are reliable.

The use of this ultrasound phantom model will bring several benefits, compared with measuring human volunteers. First, a predetermined muscle size will prove how accurately the measurement was conducted. New techniques or measurement devices can be tested on this phantom model. Second, the stabilized echogenicity may contribute to the calibration of echogenicity among different devices. Calibration is essential because echogenicity differs among devices [32]. Third, this phantom model can be used to evaluate inter-observer reliability of ultrasound measurements, especially in a multicenter study. Examiners do not need to gather in the same place to confirm the measurement correlations. This is beneficial in transport limited situations like COVID-19 outbreak. Fourth, the phantom can be used by the practice of a single person. Anyone can practice at any time with this phantom model, decreasing the infection risk of subject volunteers. Fifth, this phantom training will reduce the stress of examiners before they measure patients and of patients by reducing the scanning time. Although authors created this phantom model primarily for measuring the rectus femoris muscle, this model can be used for assessing the thickness of vastus lateralis because this phantom has a concentric semicircle structure. This phantom model has a lot of potential for its use.

This study has several limitations. First, this phantom model does not completely reflect the human subjects. The authors adjusted the ultrasound image and compression feeling, not acoustic characteristics. Therefore, our results need conscious interpretation. Second, limited information is available about the material of the phantom model. Third, the actual size of the phantom is different from the design. Thus, the phantom needs ultrasound measurements of the baseline size by well-experienced examiners. Fourth, the authors compared a different probe and device in Study 1, but the scanning setting of the ultrasound machine was not completely unified due to the technical difficulty. Fifth, the training was conducted in a limited number of volunteers, requiring further investigation. Sixth, the authors did not compare the phantom training and traditional ultrasound training without phantom. Thus, the authors cannot conclude this phantom training can be a substitute for traditional training.

## 5. Conclusions

The authors created an ultrasound phantom model for the measurement of the rectus femoris muscle. By using this model, the authors found that thickness, cross-sectional area, and echogenicity were affected by various measurement conditions. Moreover, the authors found that training various health care providers with this phantom model improved the accuracy of measurements.

## Figures and Tables

**Table 1:** Points of ultrasound measurements.

Point	Description
1	Generous amount of gel to avoid compression
2	No probe compression
3	Perpendicular vertical and horizontal probe position
4	No knee flexion (No side compression of phantom)
5	No leg rotation (No rotation of phantom)
6	Stabilized position till image capture

**Table 2:** Thickness and cross-sectional area.

		Thickness			Cross-Sectional Area		
		cm	%	*p*	cm^2^	%	*p*
Baseline		3.70 ± 0.01			7.74 ± 0.06		
(1) Probe compression strength	0.5 cm	3.59 ± 0.03	−3.0 ± 1.0	<0.01	7.58 ± 0.12	−2.0 ± 1.4	0.03
	1.0 cm	3.45 ± 0.03	−6.9 ± 0.7 †	<0.01	7.43 ± 0.12	−4.0 ± 1.8 †	<0.01
(2) Pennation angle change	10°	3.76 ± 0.03	1.6 ± 0.9 *	<0.01	8.00 ± 0.11	3.4 ± 1.0 *	<0.01
	20°	3.81 ± 0.03	2.8 ± 0.9 †	<0.01	8.21 ± 0.10	6.0 ± 1.5 †	<0.01
(3) Horizontal angle change	10°	3.70 ± 0.02	−0.1 ± 0.5 *	0.86	7.95 ± 0.14	2.7 ± 1.9 *	0.02
	20°	3.69 ± 0.02	−0.4 ± 0.8 †	0.15	8.13 ± 0.15	5.0 ± 1.8 †	<0.01
(4) Rotation of phantom	20°	3.69 ± 0.02	−0.4 ± 0.7	0.28	7.67 ± 0.13	−0.9 ± 1.7	0.30
	40°	3.70 ± 0.03	−0.1 ± 1.2	0.90	7.76 ± 0.25	0.2 ± 3.1	0.89
(5) Compression from side	weak	4.23 ± 0.01	14.3 ± 0.5 †	<0.01	7.44 ± 0.10	−3.9 ± 1.1 †	<0.01
	strong	4.55 ± 0.06	22.9 ± 1.7 †	<0.01	6.94 ± 0.09	−10.3 ± 1.5 †	<0.01
(6) Different equipment	Convex probe	3.66 ± 0.02	−1.1 ± 0.7 †	<0.01	7.87 ± 0.09	1.6 ± 0.8 †	0.04
	Different device	3.70 ± 0.02	−0.1 ± 1.0	0.75	7.68 ± 0.10	−0.8 ± 2.2	0.29

Measurements were conducted five times by a physician objectively using a scale or protractor. Percentage was the change from baseline measurement. Data were presented as mean ± standard deviation, and compared using the t-test. *p* value in this table was evaluated with the relationship between baseline measurements and measurements in various conditions. Further comparison was conducted to evaluate the percentage difference between thickness and cross-sectional area measurements, and the significance was shown with an asterisk (0.01 ≤ *p* < 0.05) or dagger (*p* < 0.01).

**Table 3:** Echogenicity in various conditions.

		Echogenicity		
		Pixel	%	*p*
Baseline		52.2 ± 0.44		
(1) Probe compression strength	0.5 cm	47.1 ± 2.31	−9.7 ± 4.6	<0.01
	1.0 cm	47.9 ± 1.24	−8.4 ± 2.4	<0.01
(2) Pennation angle change	10°	40.1 ± 1.41	−23.1 ± 2.6	<0.01
	20°	37.7 ± 3.00	−27.7 ± 5.6	<0.01
(3) Horizontal angle change	10°	51.4 ± 3.42	−1.6 ± 6.8	0.60
	20°	49.9 ± 1.97	−4.3 ± 4.1	0.04
(4) Rotation of phantom	20°	48.6 ± 0.94	−6.8 ± 1.8	<0.01
	40°	50.0 ± 1.19	−4.2 ± 1.8	<0.01
(5) Compression from side	weak	37.0 ± 2.21	−29.1 ± 4.2	<0.01
	strong	36.9 ± 2.33	−29.3 ± 4.2	<0.01
(6) Different equipment	Convex probe	57.1 ± 1.74	9.3 ± 3.8	<0.01
	Different device	40.0 ± 3.81	−23.4 ± 7.4	<0.01

Measurements were conducted five times by a physician objectively using a scale or protractor. Percentage was the change from baseline measurement. *p* value was evaluated with the relationship between baseline measurements and other variables. Data were presented as mean ± standard deviation, and compared using the *t*-test.

## Data Availability

Data are available upon reasonable request for academic, non-commercial research purposes.
